# AMP-Activated Protein Kinase Plays an Important Evolutionary Conserved Role in the Regulation of Glucose Metabolism in Fish Skeletal Muscle Cells

**DOI:** 10.1371/journal.pone.0031219

**Published:** 2012-02-16

**Authors:** Leonardo J. Magnoni, Yoryia Vraskou, Arjan P. Palstra, Josep V. Planas

**Affiliations:** Departament de Fisiologia i Immunologia, Facultat de Biologia, Universitat de Barcelona I Institut de Biomedicina de la Universitat de Barcelona (IBUB), Barcelona, Spain; Universidad Europea de Madrid, Spain

## Abstract

AMPK, a master metabolic switch, mediates the observed increase of glucose uptake in locomotory muscle of mammals during exercise. AMPK is activated by changes in the intracellular AMP∶ATP ratio when ATP consumption is stimulated by contractile activity but also by AICAR and metformin, compounds that increase glucose transport in mammalian muscle cells. However, the possible role of AMPK in the regulation of glucose metabolism in skeletal muscle has not been investigated in other vertebrates, including fish. In this study, we investigated the effects of AMPK activators on glucose uptake, AMPK activity, cell surface levels of trout GLUT4 and expression of GLUT1 and GLUT4 as well as the expression of enzymes regulating glucose disposal and PGC1α in trout myotubes derived from a primary muscle cell culture. We show that AICAR and metformin significantly stimulated glucose uptake (1.6 and 1.3 fold, respectively) and that Compound C completely abrogated the stimulatory effects of the AMPK activators on glucose uptake. The combination of insulin and AMPK activators did not result in additive nor synergistic effects on glucose uptake. Moreover, exposure of trout myotubes to AICAR and metformin resulted in an increase in AMPK activity (3.8 and 3 fold, respectively). We also provide evidence suggesting that stimulation of glucose uptake by AMPK activators in trout myotubes may take place, at least in part, by increasing the cell surface and mRNA levels of trout GLUT4. Finally, AICAR increased the mRNA levels of genes involved in glucose disposal (hexokinase, 6-phosphofructokinase, pyruvate kinase and citrate synthase) and mitochondrial biogenesis (PGC-1α) and did not affect glycogen content or glycogen synthase mRNA levels in trout myotubes. Therefore, we provide evidence, for the first time in non-mammalian vertebrates, suggesting a potentially important role of AMPK in stimulating glucose uptake and utilization in the skeletal muscle of fish.

## Introduction

AMP-activated protein kinase (AMPK) is a phylogenetically conserved enzyme which has been suggested to act as a ‘metabolic master switch’ mediating the cellular adaptation to environmental or nutritional stress factors [1]. This fuel-sensing enzyme is activated by phosphorylation when a cellular stress increases the AMP∶ATP ratio due to limited generation of ATP (e.g. hypoxia) or increased ATP depletion and, consequently, AMP production (e.g. exercise). Activation of AMPK leads to the concomitant inhibition of energy-consuming biosynthetic pathways not required for survival and to the activation of metabolic pathways that regenerate the ATP, including glucose uptake and its subsequent utilization by the tissues [2].

It is well recognized that in order to understand how energy balance is maintained in the organism it is important to study the mechanisms involved in the activation of AMPK in skeletal muscle. This organ, that contributes to 40% of the resting metabolic rate [3], undergoes an energetic challenge during exercise-induced muscle contraction, when it shows a remarkable increase in its ATP turnover rate [4]. Furthermore, AMPK is activated in the skeletal muscle of mammals by exercise and this activation is associated with an increase in glucose uptake by the tissue [5], [6]. Widespread research has been carried out studying the activation of AMPK by synthetic compounds in the mammalian muscle, using the adenosine analog 5-aminoimidiazole-4-carboxamide ribonucleoside (AICAR) and biguanide 1,1-dimethylbiguanide hydrochloride (metformin) as pharmacological tools (e.g. “exercise mimetics”) to simulate the effects of exercise on AMPK [7], [8]. Given that many fish species experience swimming-induced exercise as an integral part of their behavior and due to the fact that in fish the contractile skeletal muscle represents more than 50% of their body weight, it is conceivable that AMPK could also play a key integrative role in the physiological and metabolic adaptation to swimming in fish skeletal muscle. AMPK activity has been measured in several fish tissues, including skeletal muscle, and the enzyme appears to be regulated by phosphorylation in a manner similar to mammals [9]. More specifically, AMPK activity is up-regulated in the liver of goldfish (*Carassius auratus*) after 12 h of hypoxia [9] and hypoxic and anoxic treatments activate AMPK in the goldfish brain and cardiac muscle [10]. Recently, evidence for the pharmacological activation of AMPK has been reported for the ability of AICAR to increase AMPK activity in goldfish hepatocytes *in vitro* [11] and for both AICAR and metformin to increase AMPK activity in rainbow trout (*Oncorhynchus mykiss*) liver *in vivo* and *in vitro* [12]. However, the direct metabolic effects of compounds that activate AMPK have never been studied in fish muscle cells, despite the known importance of AMPK in glucose metabolism in mammals [13] and, specifically, in orchestrating the metabolic effects of exercise-induced contraction of locomotory muscle.

Glucose utilization is related to the ability of glucose to transfer across the plasma membrane, a step in which glucose transporters (GLUTs) play an essential role [14]. During the past few years, GLUTs expressed in skeletal muscle of mammals, mostly GLUT4, have been the focus of metabolic studies examining glucose and energy homeostasis [15]. Exercise enhances glucose transport in mammalian skeletal muscle by promoting the translocation of GLUT4 to the plasma membrane [16], [17] and by inducing a transient increase in the transcription of the GLUT4 gene in this tissue [18], [19], and both mechanisms appear to be independent of the action of insulin [20], [21]. Additionally, AMPK activators such as AICAR and metformin also increase glucose uptake in skeletal muscle through the two mechanisms involving GLUT4 [22], [23], [24]. AMPK activation causes an increase in the mRNA and protein levels of PGC-1α, a transcriptional co-factor known to have a key role regulating the expression of several genes in the mammalian muscle involved in energy metabolism, including glucose disposal [25]. However, there is no information on how AMPK activation may alter PGC-1α gene expression in fish muscle.

In the context of the prevalent notion that teleost fish, and particularly salmonid fish (i.e. trout and salmon), are relatively glucose intolerant [26], we have previously shown that, like in mammals, GLUT4 may also play a role in regulating glucose homeostasis in salmonid fish and that GLUT4 is expressed and regulated in skeletal muscle of rainbow trout and brown trout (*Salmo trutta*) in response to insulin and feeding status [27], [28], [29], [30], [31]. In particular, insulin stimulates the translocation of trout GLUT4 to the cell surface [29] as well as its mRNA and protein expression in skeletal muscle both *in vivo* and *in vitro* [28], [30], [31]. As in mammals, glucose uptake by peripheral tissues in the carp (*Cyprinus carpio*) is increased by the administration of glucose and metformin *in vivo*, improving glucose disposal [32]. Furthermore, metformin improves glucose homeostasis when infused *in vivo* in trout, an effect that is associated with increased GLUT4 expression in white muscle, suggesting a mammalian-like effect of metformin in this species [33]. In the present study, we have investigated the ability of AMPK activators to stimulate endogenous AMPK activity and glucose metabolism in trout muscle. To address this issue we have used a primary culture of brown trout muscle cells that can reproduce the differentiation process taking place in skeletal muscle [31] and that we have previously used to study the direct metabolic effects of hormones and cytokines in trout muscle [29], [31], [34]. The results from the present study indicate that the AMPK activators AICAR and metformin increase AMPK activity in trout myotubes, resulting in an increase in GLUT4-mediated glucose uptake and possibly also utilization, and suggest that AMPK may play an important metabolic role in fish skeletal muscle, particularly under conditions during which energy expenditure is increased (e.g. exercise).

## Materials and Methods

### Animals

Brown trout (*Salmo trutta*) weighing 8–20 g were supplied by the Piscifactoria de Baga (Generalitat de Catalunya, Baga, Spain). Animals were maintained in the facilities of the School of Biology at the University of Barcelona in a closed-water flow circuit with water at a temperature of 14°C. They were fed *ad libitum* with a commercial diet and fasted 24 h prior to the experiments. The experimental protocols used for trout in this study have been reviewed and approved by the Ethics and Animal Welfare Committee of the University of Barcelona, Spain.

### Isolation and culture of trout muscle cells

Animals were sacrificed by a blow to the head and the skin was sterilized by immersion in 70% ethanol for 30 seconds. Trout muscle cell isolation and culture was performed as initially described by Fauconneau and Paboeuf [35] with some modifications [30], [31], [34]. This cell system has been widely used and validated by our group and others to study hormone and cytokine action in undifferentiated and differentiated trout muscle cells *in vitro* [29], [31], [34], [36]. Briefly, the skin was removed and dorsal white muscle was isolated in sterile conditions and collected in DMEM at pH 7.4, containing 9 mM NaHCO_3_, 20 mM Hepes, 15% horse serum, antibiotic-antimycotic cocktail (100 U/ml penicillin, 100 µg/ml streptomycin, 25 µg/ml amphotericin B) and 0.15% gentamycin. The tissue was mechanically dissociated and then enzymatically digested with a 0.2% collagenase solution in DMEM for 1 h at 18°C and gentle shaking. After centrifugation (300 g for 10 min at 15°C), the resulting pellet was washed in DMEM without horse serum and was enzymatically digested twice with a 0.1% trypsin solution in DMEM for 20 min at 18°C with gentle shaking. The resulting cellular suspension was filtered through 100 and 40 µm nylon filters. The obtained cells were counted and plated on 12-well plates (BD Biosciences, Madrid, Spain) at a density of 1.8–2×10^6^ cells/well. Plates were treated the day before with poly-L-lysine and laminin to facilitate cell adhesion and subsequent differentiation. Trout muscle cells were maintained at 18°C in DMEM containing 9 mM NaHCO_3_, 20 mM Hepes, 10% FBS and antibiotic-antimycotic cocktail (100 U/ml penicillin, 100 µg/ml streptomycin, 25 µg/ml amphotericin B). After 24 h of plating, plates were washed to eliminate non-adherent cells. Media was routinely renewed every second day, while cultures were monitored by daily observation under an inverted microscope (Zeiss Axiovert 25). Trout muscle cells were cultured until they spontaneously differentiated into myotubes after 8–10 days [31]. Media, enzymes and reagents used during the isolation and culture were purchased from Sigma-Aldrich (Tres Cantos, Spain).

### Glucose uptake measurements in trout muscle cells

In order to determine the effects of AMPK activators on glucose transport in myotubes, cells were incubated in the absence (Control) or in the presence of AICAR (Sigma-Aldrich; 50, 250 and 500 µM) or metformin (Sigma-Aldrich; 10, 100 and 500 µM). The specific doses and incubation times used in the current study have been optimized based on previous dose- and time-trials. Recombinant human insulin (Sigma-Aldrich) was added at a concentration of 1 µM to the cells for 30 min prior to the glucose uptake assays as a positive control, as previously described [31], [34], and to study its possible synergistic action with the AMPK activators. In addition, 6-[4-(2-Piperidin-1-ylethoxy) phenyl]-3-pyridin-4-ylpyrazolo [1,5-a] pyrimidine (Compound C; Sigma-Aldrich), a protein kinase inhibitor widely used to inhibit AMPK, was added to a final concentration of 40 µM in the presence of the AMPK activators or alone. AMPK activators and Compound C were applied directly to cells plated in 12-well plates for 24 h after repeatedly washing the cells with DMEM to remove the growth media.

Determination of 2-deoxyglucose (2-DG) uptake in trout muscle cells was performed as previously described [29], [31]. Briefly, cells were washed twice with HEPES-buffered saline and were incubated with the same buffer containing 50 µM 2-DG (0.5 µCi/ml 2-[^3^H]-DG) for 30 min at 18°C. Subsequently, cells were rinsed three times with ice-cold PBS solution (154 mM NaCl, 5.6 mM Na_2_HPO_4_, 1.1 mM KH_2_PO_4_) containing 50 mM D-glucose. Finally, cells were lysed with 0.05 N NaOH and lysates were counted with scintillation liquid in a β-counter (Packard Bioscience, Meriden, CT). Nonspecific uptake was carried out in the presence of cytochalasin B (50 µM) during the assay, and these values were subtracted from all other values. Protein content in the lysates was measured using the Bio-Rad Protein Assay kit (Bio-Rad, Barcelona, Spain). Glucose uptake was measured in triplicate, normalized to total protein and expressed as fold induction with respect to non-stimulated cells.

The viability of trout myotubes was assessed by determining LDH activity released into the culture media of myotubes incubated for 24 h with AMPK activators and the AMPK inhibitor as described above. LDH activity was measured using a commercial kit (Spinreact, Sant Esteve de Bas, Girona, Spain) following the manufacturer's instructions. No statistically significant differences in the levels of LDH activity in the media were found when myotubes were exposed for 24 h to AICAR, metformin and/or Compound C with respect to control values (data not shown).

### Culture and cell surface labeling of L6 cells expressing brown trout GLUT4

In order to determine if the cell surface levels of brown trout GLUT4 increase in response to AMPK activators in skeletal muscle cells, we used a rat skeletal muscle cell line L6 stably expressing brown trout GLUT4 harboring an exofacial myc epitope (btGLUT4myc) that has been used previously by our group to study the traffic characteristics of brown trout GLUT4 [29]. Briefly, L6-btGLUT4myc myoblasts were maintained in α-MEM supplemented with 10% FBS and 1% antibiotic/antimycotic solution (100 U/ml penicillin, 100 µg/ml streptomycin, 25 µg/ml amphotericin B) in a humidified atmosphere of air and 5% CO_2_ at 37°C. Myotubes obtained by differentiating L6-btGLUT4myc myoblasts in media supplemented with 2% FBS within five days after seeding were serum-starved for two hours and subsequently treated with AICAR (2 mM) or metformin (1 mM) for 18 h. The doses of the AMPK activators used for these experiments were chosen based of the effective doses reported in the literature to affect GLUT4 traffic in mammalian cells [37], [38]. As a positive control, cells were treated for 20 min with 100 nM human insulin, which stimulates btGLUT4 translocation in this cell line [29]. Media was removed and cells were washed three times with ice-cold PBS supplemented with 1 mM CaCl_2_ and 1 mM MgCl_2_ at 4°C (PBS+, pH 7.4). Cells were then fixed with 3% paraformaldehyde for 15 min on ice and quenched with 100 mM glycine for 10 min. To label cell surface btGLUT4myc, cells were blocked in 5% goat serum in PBS+ for 10 min, and then incubated with α-myc antibody solution (1.0 µg/ml in PBS+ with 5% goat serum) for 1 h at room temperature. Following labeling, excess anti-myc antibodies were removed by extensive washing in ice-cold PBS+. Cell surface GLUT4-bound anti-myc antibodies were probed with HRP-conjugated secondary antibodies followed by detection of bound HRP by the colorimetric o-phenylenediamide assay, as previously described [39]. The fraction of btGLUT4myc on the cell surface was measured in triplicate and expressed as fold induction with respect to non-stimulated cells and normalized to total protein.

### Determination of AMPK activity

In order to detect the activation of endogenous AMPK by synthetic compounds, trout myotubes were incubated in the absence or presence of AICAR (250 µM) or metformin (10 µM) for 24 h at the doses observed to stimulate glucose uptake in this cell system. Cells were washed and scraped from the plates with PBS and lysates were obtained using ice-cold RIPA buffer in the presence of protease (10 µl/ml) and phosphatase inhibitor cocktails (10 µl/ml), according to the manufacturer's instructions (Sigma-Aldrich). Protein content in the lysates was measured using the Bio-Rad Protein Assay kit (Bio-Rad). AMPK activity in lysates from trout myotubes was determined using the CycLex AMPK Kinase Assay Kit (CycLex Co., Ltd., Nagano, Japan). Briefly, samples were incubated for 30 min at 30°C in the presence of ATP (50 µM) in a plate pre-coated with a substrate peptide, corresponding to mouse IRS-1. AMPK activity in cell lysates was measured by monitoring the phosphorylation of Ser-789 in IRS-1 using an anti-mouse phospho-Ser-789 IRS-1 monoclonal antibody and peroxidase-coupled anti-mouse IgG antibody. Conversion of the chromogenic substrate tetra-methylbenzidine was quantified by measuring changes in absorbance at 450 nm. AMPK activity (specific kinase activity) was calculated as the difference between the absorbance measured in the absence or in the presence of Compound C (10 µM). Activity was measured in duplicates, normalized to total protein in the cell lysates, and expressed as fold induction with respect to non-stimulated cells.

### Identification of AMPK subunits in trout skeletal muscle cells by Western blotting

Western blot analyses were conducted using lysates from trout myotubes (as described above) and from C2C12 myotubes, used as a positive control. Myotube lysates (20–40 µg protein) were diluted in Laemmli sample buffer, heated for 5 min at 95°C, and centrifuged at 12000 g for 5 min. Proteins from the supernatant and protein standards (Precision Plus, BioRad, Spain) were loaded and separated on 12% SDS-PAGE precast gels using a Mini-Protean system (BioRad, Spain) for 1–2 h at 100 V and then transferred to a polyvinylidene difluoride (PVDF) membrane (Millipore, Madrid, Spain). The membrane was blocked with blocking buffer (20 mM Tris-HCl, 150 mM NaCl, 0.05% Tween-20, pH 7.6) containing 5% (w/v) non-fat dry milk for 1 h. The membrane was washed several times in blocking buffer and incubated overnight at 4°C with rabbit antibodies against the different subunits of human AMPK (α_1_, α_2_, β_1_, β_2_, γ_1_, γ_2_ and γ_3_) AMPK Subunit Antibody Sampler Kit, Cell Signaling, Barcelona, Spain), diluted to 1∶1000 in blocking buffer containing 5% Bovine Serum Albumin (BSA, w/v) under continuous shaking. After three washes, the membrane was incubated with a secondary antibody against rabbit IgG conjugated with horseradish peroxidase (Cell Signaling, Barcelona, Spain) diluted 1∶2000 in TBST buffer containing 5% BSA. Immune complexes were detected using a chemiluminescent substrate (Supersignal West Pico, ThermoScientific, Spain) according to the manufacture's instructions and visualized with a luminescent image analyzer (FujiFilm LAS-3000).

### Determination of glycogen content

To investigate the effect of AMPK activators on glycogen storage, trout myotubes were incubated with AICAR (250 µM), metformin (10 µM) or media alone (Control) for 24 h. Cell lysates were obtained as described in the previous section and used to assay glycogen content by the amyloglucosidase hydrolysis method [40]. The amount of glucose obtained after glycogen breakdown in trout myotube lysates, after subtracting free glucose levels, was measured in triplicate using a commercial assay kit (SpinReact, Spain), and normalized to the total amount of protein in the cell lysates.

### Quantification of gene expression by qPCR

In order to test the effects of AMPK agonists on the expression of genes regulating glucose utilization, including glucose transporters (GLUT1 and GLUT4), key enzymes in glucose metabolism (hexokinase (HK), 6-phosphofructokinase (6-PFK), pyruvate kinase (PK), glycogen synthase (GS), citrate synthase (CS)), and the transcriptional coactivator peroxisome proliferator-activated receptor γ coactivator 1α (PGC-1α, trout myotubes were incubated with AICAR (250 µM), metformin (10 µM) or media alone (Control). After a 24 h incubation, total RNA was extracted from cultured myotubes using Trizol reagent (Invitrogen, Barcelona, Spain), and reverse-transcribed to cDNA using SuperScript III Transcriptase (Invitrogen), oligo(dT) primer and random hexamer primers (Promega, Barcelona, Spain), according to the manufacturer's protocols. Primer sequences, amplicon sizes and GenBank accession numbers of the target genes are presented in Table 1. For the amplification of trout 6-PFK, GS, CS and PGC-1α genes, primers were designed using the Genamics Expression software. Primers for the amplification of trout glucose transporters GLUT1 and GLUT4 utilized in this study were previously reported by Diaz et al. [31] and Planas et al. [27], respectively. Primers used for the amplification of HK and PK were reported by Panserat et al. [41]. The qPCR reactions contained 10 µl of SYBR GreenER qPCR SuperMix (Invitrogen), 500 nM of forward and reverse primers, 3 µl RNase/DNase-free water and 5 µl of template DNA (cDNA at a 1∶25 dilution or plasmid DNA), in a final volume of 20 µl. The reactions were run in a MyiQ Real-Time PCR Detection System (Bio-Rad) using the following protocol: 2 min at 50°C, 8 min at 95°C, followed by 40 cycles of 15 sec denaturation at 95°C and 30 sec at the temperature included in Table 1 for each pair of primers, and a final melting curve of 81 cycles from 55°C to 95°C (0.5°C increments every 10 sec). All the samples were run in triplicate and fluorescence was measured at the end of every extension step. Fluorescence readings were used to estimate the Ct values used to calculate the number of copies of the gene of interest. Changes in HK, 6-PFK, GS, CS and PGC-1α expression were analyzed using the quantification method by qPCR described by Livak and Schmittgen [42]. In this method, values for each sample were expressed as fold change, calculated relative to control group and normalized for each gene against 18S ribosomal RNA as the reference gene. Expression of 18S was not affected by any of the treatments (data not shown). Changes in GLUT1 and GLUT4 gene expression by qPCR was analyzed according to the method described by Whelan et al. [43]. In this method, the Ct values were used to calculate copy numbers with standard plots constructed for each target gene using specific primers and serial dilutions (10^−4^ to 10^−9^) of plasmid DNA corresponding to cloned PCR products into the pGEM-T Easy vector (Promega), which was previously quantified using a QubitTM fluorometer (Invitrogen). The Ct values were plotted against the logarithm of their initial template copy numbers, generating a standard curve by linear regression of the plotted points that are utilized for the calculations. All the standard curves exhibited correlation coefficients higher than 0.99, and efficiencies were greater than 99%.

**Table 1:** Sequences of primers used in gene expression analyses by qPCR (F: Forward; R: Reverse).

GenBank	Gene name	Primer sequence (5′-3′)	Amplicon size (bp)
AF247728	GLUT1	(F) AATATCGACAAGCCACGCTG	270
		(R) GAGAAGGAGCCGAAGATACC	
AF247395	GLUT4	(F) GTGCCAGGCTTATTGTCCATATTC	357
		(R) TAGAGAAGATGGCTACCGACAG	
AY864082.1	HK	(F) CTGGGACGCTGAAGACCAGA	159
		(R) CGGTGCTGCATACCTCCTTG	
AM083785	6-PFK	(F) GATGGGAGTGGAGGCTGTGAT	239
		(R) CTGGAGGGTTGATGTGGGCTA	
AF246146.1	PK	(F) GTCCAATGACCCTACTGAGG	306
		(R) CCTGTCTTGAAGAAGCCCCT	
BT073381.1	GS	(F) CGTGGTGAGAGGAAGGAACTGAGC	236
		(R) CCGTTGAGACCGTGGAGACA	
TC89195	CS	(F) CAACCAACCTCACTCATCACCATA	133
		(R) GCAGCAGAAGCAGCCCATAA	
CA368123	PGC-1α	(F) CTCCAAGCACTACAAAAACCCAACT	127
		(R) CACCACATCCCAAACACCAGA	
AF308735	18S	(F) CGGAGGTTCGAAGACGATCA	62
		(R) TCGCTAGTTGGCATCGTTTAT	

### Statistical analysis

Values are given as mean ± SE. Analysis of differences between groups was performed by the Mann–Whitney Rank Sum Test and were considered statistically significant when *P*<0.05.

## Results

### Identification of AMPK subunits in trout myotubes

**Figure 1:**
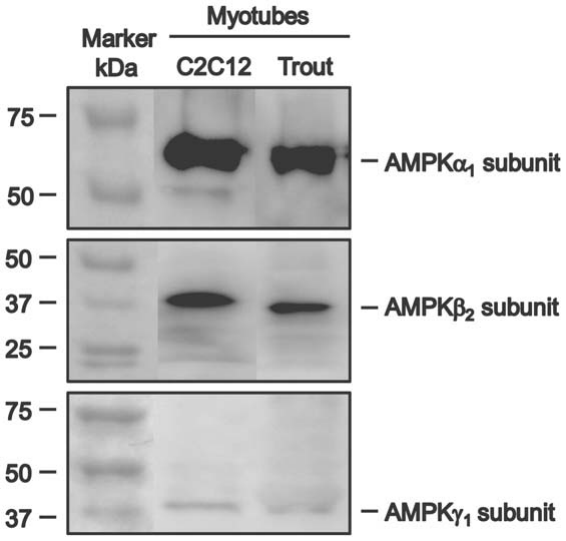
Immunodetection of AMPK subunits in trout myotubes. Western blots were performed with lysates from trout myotubes and from C2C12 myotubes, used as a positive control, subjected to SDS-PAGE (12%) and blotted onto PVDF membranes, using commercial antibodies (at 1∶1000) against the α_1_, β_2_ and γ_1_ subunits of human AMPK. The molecular weight of the protein markers is expressed in kDa.

In order to identify the presence of the AMPK heterotrimeric (α, β, γ) protein complex in the skeletal muscle of trout, antibodies to the catalytic (α_1_ and α_2_) and regulatory subunits (β_1_, β_2_, γ_1_, γ_2_ and γ_3_) of human AMPK were used for Western blotting with lysates from trout myotubes. As shown in Figure 1, immunoreactive proteins of approximately 63 kDa, 37 kDa and 40 kDa were detected in trout myotubes with antibodies to the α_1_, β_2_ and γ_1_ subunits of AMPK, respectively. As a positive control, these antibodies also detected the α_1_, β_2_ and γ_1_ AMPK subunits in C2C12 myotubes at their expected molecular sizes, which were similar to the molecular sizes of the three trout proteins identified. However, we were unable to detect the α_2_, β_1_, γ_2_ and γ_3_ subunits of AMPK in trout myotubes, although they were all present in the positive control (data not shown).

### AMPK activators stimulate glucose uptake in trout myotubes

**Figure 2:**
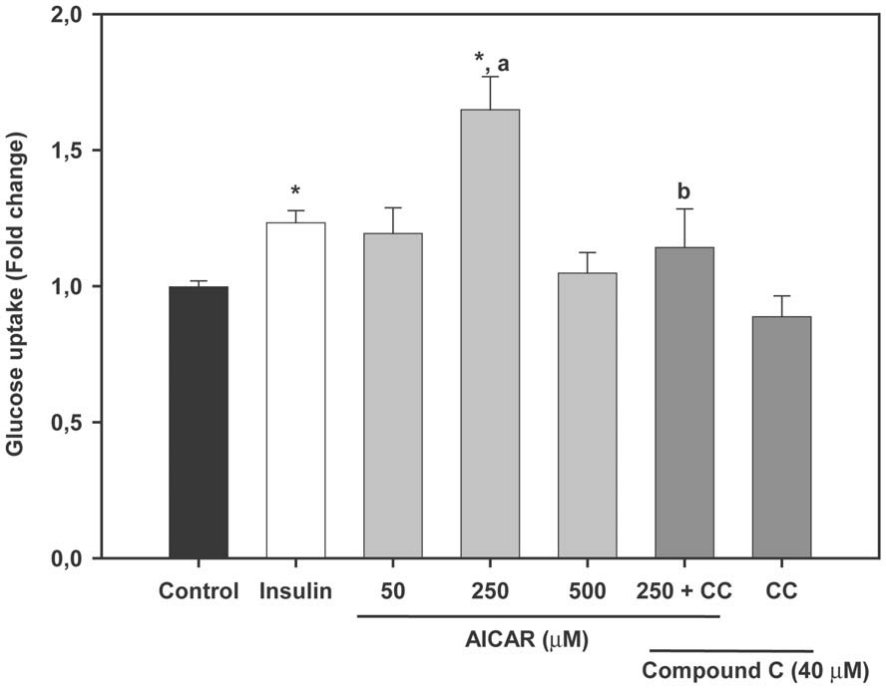
Effects of AICAR on glucose uptake in trout myotubes. Cells were serum starved and stimulated for 24 h in the absence or presence of different doses of AICAR (50, 250 and 500 µM). The effects of AICAR (250 µM) we also tested in the absence or presence of Compound C (CC, 40 µM). Insulin was used as a positive control (1 µM) during the last 30 min of the incubation. Glucose uptake was subsequently determined as described in Materials and Methods. Results are expressed as fold stimulation above the control group, which was set to 1, and shown as the means ± SE of six independent experiments, each performed in triplicate. The asterisk indicates significant differences from control, where different letters indicate significant differences between treatment with or without the inhibitor (P<0.05).

**Figure 3:**
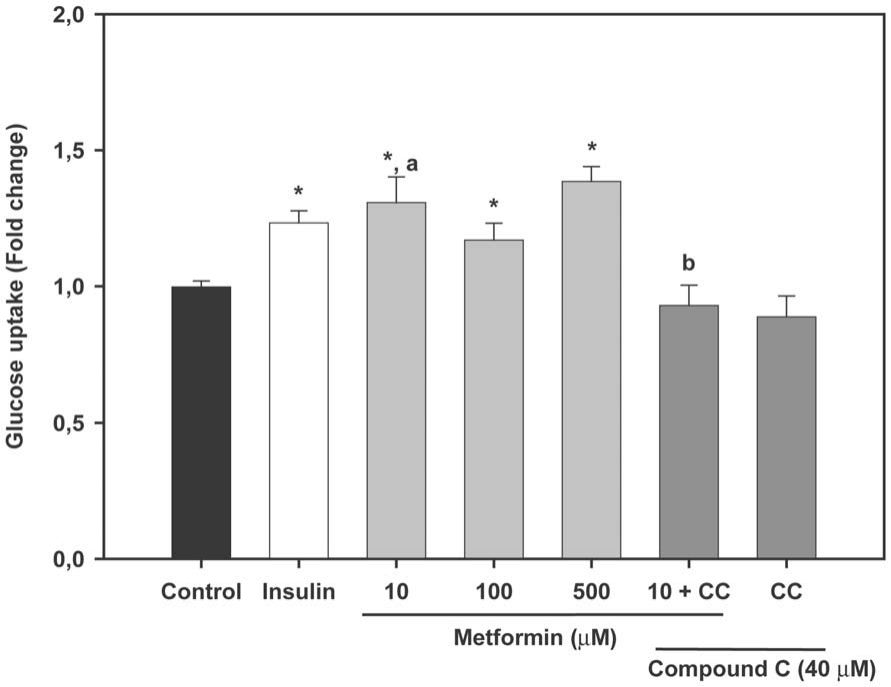
Effects of metformin on glucose uptake in trout myotubes. Cells were serum starved and stimulated for 24 h in the absence or presence of different doses of metformin (10, 100 and 500 µM). The effects of metformin (10 µM) we also tested in the absence or presence of Compound C (CC, 40 µM). Insulin was used as a positive control (1 µM) during the last 30 min of the incubation. Glucose uptake was subsequently determined as described in Materials and Methods. Results are expressed as fold stimulation above the control group, which was set to 1, and shown as the means ± SE of six independent experiments, each performed in triplicate. The asterisk indicates significant differences from control, where different letters indicate significant differences between treatment with or without the inhibitor (P<0.05).

As a first step to investigate the possible role of AMPK in glucose metabolism in fish skeletal muscle, we examined the effects of two well-described AMPK activators in mammals (i.e. AICAR and metformin) on glucose uptake in trout myotubes. We first incubated the cells for 24 h in the presence or absence of different doses of AICAR (50, 250 and 500 µM) and observed that this compound significantly stimulated glucose uptake in myotubes only at a dose of 250 µM (1.6 fold, P<0.01) (Fig. 2). Similarly, incubation of trout myotubes with metformin (at doses of 10, 100 and 500 µM) resulted in a statistically significant stimulation of glucose uptake at all doses tested (1.3, 1.2 and 1.4 fold respectively; P<0.05) (Fig. 3). AICAR and metformin (at their maximally effective doses) were equally or even more potent than insulin in stimulating glucose uptake by trout myotubes. Therefore, our results suggest that AMPK activators shown to stimulate glucose uptake in mammalian muscle cells are also effective in stimulating glucose uptake in trout myotubes. Phenformin, another biguanide known to increase glucose uptake in mammalian skeletal muscle cells by indirectly increasing AMPK activity, also stimulated glucose uptake in brown trout myotubes (data not shown). Therefore, these observations led us to hypothesize that the AMPK activators tested may have stimulated glucose uptake by increasing AMPK activity in trout muscle cells.

### AMPK activators combined with insulin do not increase further glucose uptake in trout myotubes

**Figure 4:**
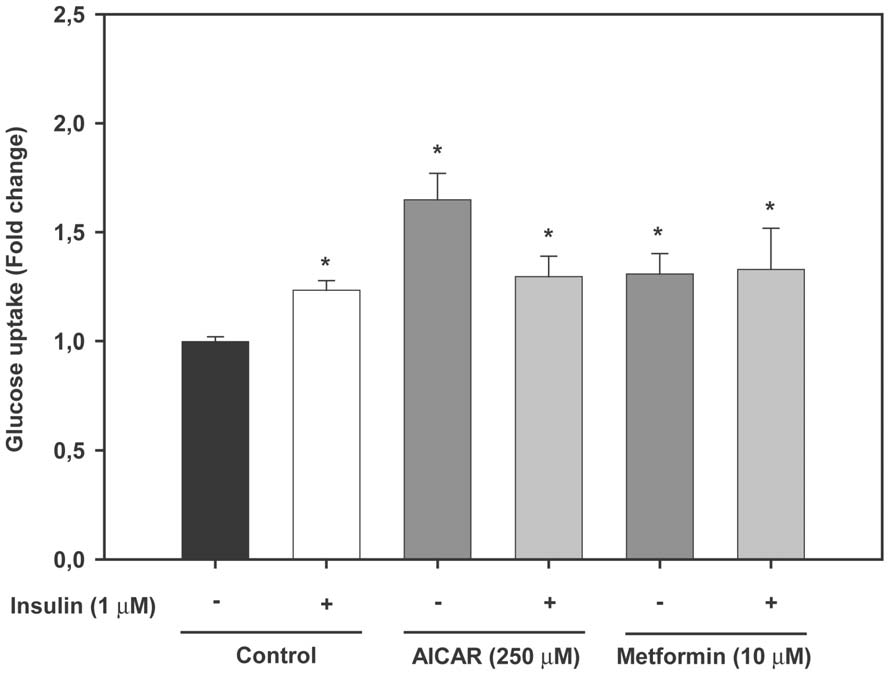
Effects of AMPK activators and insulin on glucose uptake in trout myotubes. Cells were serum starved and stimulated for 24 h in the absence or presence of different doses of AICAR (250 µM) or metformin (10 µM) and with or without insulin (1 µM), which was added during the last 30 min of the incubation. Glucose uptake was subsequently determined as described in Materials and Methods. Results are expressed as fold stimulation above the control group, which was set to 1, and shown as the means ± SE of six independent experiments, each performed in triplicate. The asterisk indicates significant differences from control (P<0.05).

In order to examine if AMPK activators had an additive or synergistic effect with insulin on glucose uptake by trout myotubes, cells were incubated with AICAR (250 µM) or metformin (10 µM) for 24 h in the presence or absence of insulin (1 µM) during the last 30 min of the incubation, as we had shown in previous studies that glucose uptake in trout myotubes was stimulated by insulin under such conditions [30], [31]. We observed that the combined presence of AMPK activators and insulin did not significantly stimulate glucose uptake in trout myotubes when compared to the values obtained when cells were incubated with the same AMPK activators alone (Fig. 4). These results indicate that AMPK activators and insulin do not have additive or synergistic effects on glucose uptake in trout muscle cells.

### Compound C abrogates the stimulatory effects of AMPK activators on glucose uptake in trout myotubes

In order to provide evidence for the involvement of AMPK activity in the stimulatory action of AMPK activators on glucose uptake in trout myotubes, we incubated the cells with AICAR or metformin in the presence of Compound C, a protein kinase inhibitor that inhibits AMPK activity [8] and that has been shown to be a useful tool to study the function of AMPK in skeletal muscle cells [44]. In support of the hypothesis that AMPK activators may have stimulated glucose uptake by increasing AMPK activity in brown trout myotubes, Compound C (40 µM) caused a complete and significant (P<0.05) inhibition of the stimulatory effects of AICAR (250 µM) and metformin (10 µM) on glucose uptake (Figs. 2 and 3). Compound C alone did not have any effect on basal glucose uptake (Figs. 2 and 3), suggesting that AMPK activity may not be required for the entry of glucose into trout myotubes under basal, non-stimulated conditions.

### AMPK activators increase AMPK activity in trout myotubes

**Figure 5:**
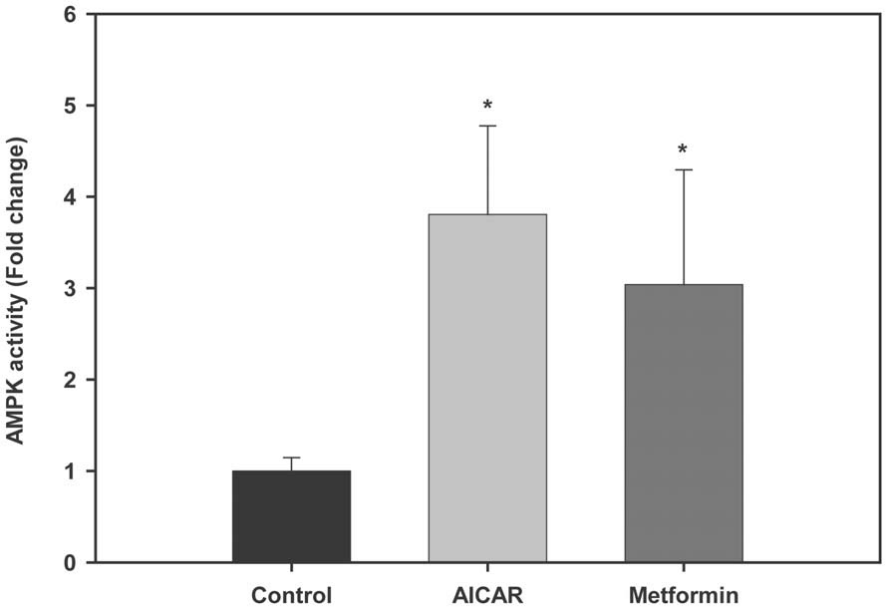
Effects of AMPK activators on AMPK activity in trout myotubes. Cells were serum starved and stimulated for 24 h in the absence or presence of AICAR (250 µM) or metformin (10 µM). AMPK activity was subsequently determined in the cell lysates as described in Materials and Methods. Results are expressed as fold stimulation above the control group, which was set to 1, and shown as means ± SE of five independent experiments, each performed in duplicate. The asterisk indicates significant differences from control (P<0.05).

To provide further evidence for the possible involvement of AMPK in the action of AMPK activators on glucose uptake, we analyzed the effects of AICAR and metformin on the activity of AMPK in trout myotubes at the doses shown to significantly stimulate glucose uptake (250 µM and 10 µM, respectively; Figs. 2 and 3). We observed that the activity of AMPK measured in brown trout myotubes was significantly increased in the presence of AICAR (3.8 fold) or metformin (3 fold) when compared to that measured in control, untreated myotubes (P<0.05, Fig. 5).

### AMPK activators increase the cell surface levels of trout GLUT4 in btGLUT4myc-expressing L6 cells

**Figure 6:**
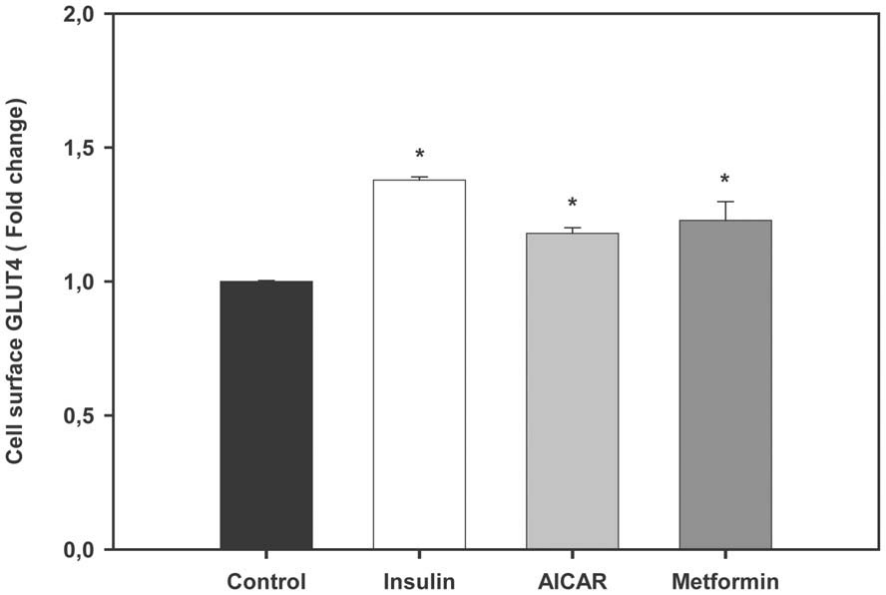
Effects of AMPK activators on trout GLUT4 cell surface levels in btGLUT4myc L6 cells. Cells were serum starved and stimulated for 18 h in the absence or presence of AICAR (2 mM) or metformin (1 mM). Insulin (100 nM), used as a positive control, was added during the last 20 min of the incubation. The proportion of trout GLUT4myc at the cell surface relative to control was determined as described in Materials and Methods. Results are expressed as fold stimulation above the control group, which was set to 1, and shown as means ± SE of six independent experiments, each performed in triplicate. The asterisk indicates significant differences from control (P<0.05).

In view of the stimulatory effects of AMPK activators on glucose uptake by trout myotubes and on the well-known stimulation of GLUT4 translocation by AMPK activators in mammalian muscle cells [45], [46], we hypothesized that AMPK activators may have stimulated glucose uptake by modulating the cell surface levels of brown trout GLUT4. In order to test this hypothesis, for lack of a fish cell line, we used a mammalian L6 muscle cell line generated by our group that stably expresses brown trout GLUT4 with an exofacial myc epitope (btGLUT4myc) and that has been a useful tool to study the traffic of trout GLUT4 in muscle cells [29], [34]. Using this cell line, we have previously reported that the cell surface levels of btGLUT4 increase in response to extracellular factors (e.g. insulin and tumor necrosis factor alpha) that stimulate glucose uptake in trout muscle cells [29], [34]. Our results indicate that btGLUT4myc L6 cells incubated in the presence of AICAR (2 mM; a dose shown to increase the cell surface levels of rat GLUT4 in ratGLUT4myc L6 cells; [38]) and metformin (1 mM) for 18 h displayed a modest but statistically (P<0.001) significant increase (1.2 fold) in the amount of btGLUT4 at the cell surface (Fig. 6).

### AMPK activators increase GLUT4, but not GLUT1, mRNA levels in trout myotubes

**Figure 7:**
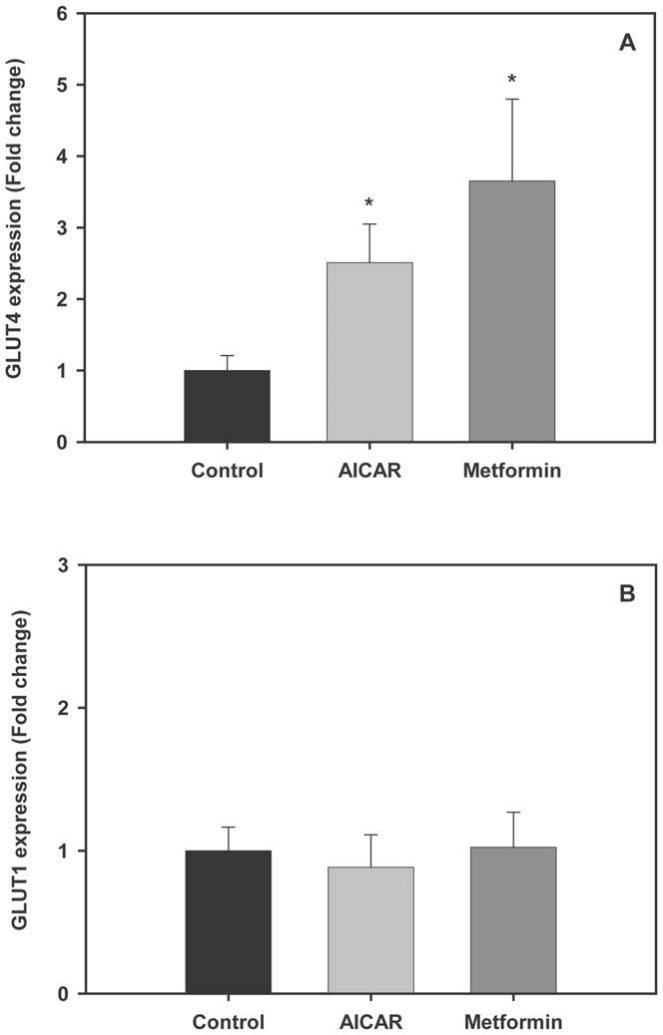
Effects of AMPK activators on GLUT4 and GLUT1 expression in trout myotubes. Cells were serum starved and stimulated for 24 h in the absence or presence of AICAR (250 µM) or metformin (10 µM). Total RNA from trout myotubes was isolated and reverse transcribed to cDNA. GLUT4 (A) and GLUT1 (B) mRNA expression levels were determined by absolute qPCR quantification as described in Materials and Methods. Results are expressed as fold stimulation above the control group, which was set to 1, and shown as means ± SE of five independent experiments, each performed in triplicate. The asterisk indicates significant differences from control (P<0.05).

In order to determine the effects of AMPK activators on glucose transporter expression in brown trout muscle cells, we investigated the effects of AICAR (250 µM) and metformin (10 µM) on the mRNA levels of GLUT1 and GLUT4, two genes known to be expressed in trout skeletal muscle and to be regulated under nutritional and hormonal manipulation [27], [28], [31]. Trout myotubes incubated for 24 h with AICAR or metformin displayed significantly higher GLUT4 mRNA levels with respect to untreated myotubes (2.5 and 3.6 fold, respectively, Figure 7A, P<0.05). However, the mRNA levels of GLUT1 in trout myotubes were not affected by any of the AMPK activators tested (Fig. 7B).

### AMPK activators do not alter glycogen content in trout myotubes

**Figure 8:**
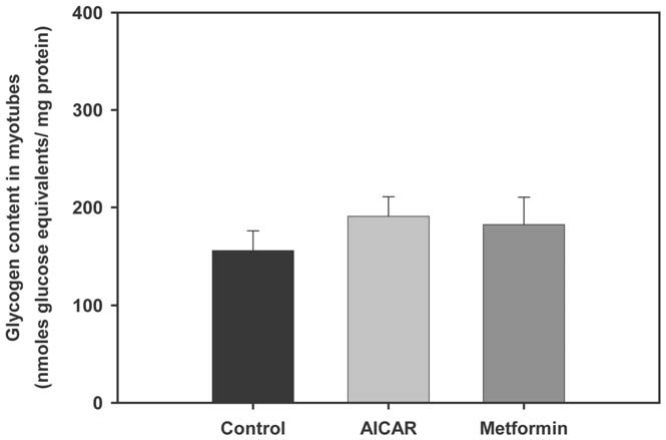
Effects of AMPK activators on glycogen content in trout myotubes. Cells were serum starved and stimulated for 24 h in the absence or presence of AICAR (250 µM) or metformin (10 µM). Glycogen content was subsequently determined in the cell lysates as described in Materials and Methods. Results are expressed as nmoles of glucose released from glycogen hydrolysis per mg of protein, and shown as means ± SE of five independent experiments, each performed in triplicate.

In view of the stimulatory effects of AMPK activators on glucose uptake and on the possible involvement of GLUT4 in this process in trout myotubes, we investigated if these effects were accompanied by changes in the amount of glycogen stored in those cells. Our results indicate that glycogen content was not affected by AMPK activators since the levels of glycogen stored in cells treated with AICAR (250 µM) or metformin (10 µM) were not significantly different than in non-treated trout myotubes (Fig. 8).

### AICAR increases the mRNA levels of genes involved in glucose disposal, generation of energy, and transcriptional regulation in trout myotubes

**Figure 9:**
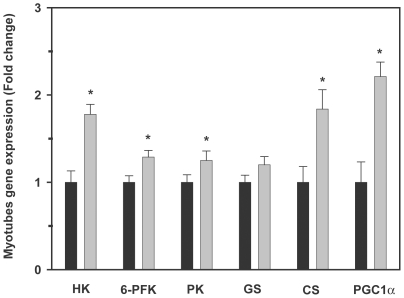
Effects of AICAR on hexokinase (HK), 6-phosphofructokinase (6-PFK), pyruvate kinase (PK), glycogen synthase (GS), citrate synthase (CS), and peroxisome proliferator-activated receptor gamma coactivator 1-alpha (PGC-1α) mRNA expression levels in trout myotubes. Cells were serum starved and stimulated for 24 h in the absence or presence of AICAR (250 µM). Total RNA from trout myotubes was isolated and reverse transcribed to cDNA, and mRNA expression levels were determined by qPCR as described in Materials and Methods. Results are expressed as fold stimulation above the control group, which was set to 1, and shown as means ± SE of five independent experiments, each performed in triplicate. The asterisk indicates significant differences from control (P<0.05).

In order to further investigate the possible fate of glucose taken up by trout myotubes as a result of the pharmacological activation of AMPK, given the similar effects of AICAR and metformin on all other parameters examined, we evaluated the effects of AICAR alone on the expression of several genes involved in glucose utilization and the generation of energy, including HK (glucose phosphorylation), 6-PFK and PK (glycolysis), GS (glycogen synthesis) and CS (tricarboxylic acid (TCA) cycle). Furthermore, we studied the effects of AICAR on the mRNA levels of PGC-1α a transcriptional coactivator that is critical in the regulation of the metabolism of the cell. Trout myotubes incubated for 24 h with AICAR displayed significantly higher mRNA levels of HK, 6-PFK, PK, CS and PGC-1α with respect to untreated myotubes (1.8, 1.3, 1.2, 1.8 and 2.2 fold, respectively; Fig. 9). However, consistent with the lack of change in glycogen levels, the mRNA levels of GS in trout myotubes were not affected by AICAR (Fig. 9).

## Discussion

### Identification of the AMPK heterotrimeric complex in trout skeletal muscle cells

AMPK, an enzyme with a key role in energy sensing and in the regulation of energy balance, is a heterotrimeric protein complex composed of a catalytic α subunit and two regulatory subunits: β and γ [47]. In order to investigate the potential role of AMPK in regulating glucose metabolism in the skeletal muscle of fish, we first set out to characterize the nature of the AMPK complex in trout myotubes. Here we report for the first time on the identification of AMPK complex; namely, the α_1_, β_2_ and γ_1_ protein subunits of trout AMPK in skeletal muscle cells, corresponding to proteins of approximately 63, 37 and 40 kDa in molecular weight that likely represent the α_1_, β_2_ and γ_1_ subunits cloned recently in rainbow trout [12].

### AMPK may increase glucose uptake through a GLUT4-mediated mechanism

From a functional point of view, in this study we report for the first time in fish that pharmacological activation of endogenous AMPK results in the stimulation of glucose uptake in skeletal muscle cells. These results are in agreement with the well established effects of AICAR and metformin as AMPK activators in skeletal muscle of mammals, which cause a concomitant increase in glucose uptake by the tissue [48], [49]. In particular, the adenosine analog AICAR increases glucose uptake in rat skeletal muscle without large changes in ATP, ADP, or AMP concentrations [22], [23]. AICAR enters the cells by adenosine transporters and is phosphorylated by adenosine kinase to ZMP, a mono-phosphorylated derivative [50]. The increase of ZMP activates AMPK [51], mimicking the multiple effects of AMP on the enzyme, including its activation by allosteric regulation and phosphorylation [52]. Incidentally, high levels of ZMP have been reported to inhibit AMPK activity [51] which could explain the lack of stimulatory effects of the highest dose of AICAR (500 µM) tested on glucose uptake in trout myotubes. On the other hand, metformin is believed to increase AMPK activity by inhibiting mitochondrial ATP synthesis and, therefore, by increasing cellular AMP∶ATP ratios [1]. Therefore, the stimulation of glucose uptake in trout myotubes by two different and well-known AMPK activators that operate through different mechanisms suggests that they may activate endogenous AMPK in fish skeletal muscle cells. Indeed, we demonstrate here that treatment of trout myotubes with AICAR or metformin results in a significant increase in AMPK activity, as in mammalian muscle cells [8], [53]. Further support for the hypothesis that AMPK activators may stimulate glucose uptake in brown trout myotubes by activating endogenous AMPK is derived by our results on the ability of Compound C, a broadly used inhibitor of AMPK that binds to the ATP-binding site on the enzyme [8], to completely block the effects of AICAR and metformin on glucose uptake. It should be mentioned, however, that despite it is well established in the literature that AICAR effectively activates AMPK, this compound is known to have effects on several non protein kinase targets [47], which would lead to the possibility that AICAR may stimulate glucose uptake in our cell system through mechanism(s), at least in part, independent of AMPK. In the present study, several observations support our hypothesis that the stimulatory effects of AICAR on glucose uptake in trout myotubes are likely mediated by AMPK. First, metformin, an unrelated compound that activates AMPK through a completely different mechanism than AICAR, has similar effects to AICAR on glucose uptake. Second, Compound C, a broadly used AMPK inhibitor, completely blocked the stimulatory effects of AICAR (and metformin) on glucose uptake. Finally, AICAR and metformin both stimulated AMPK activity in trout myotubes. Furthermore, in addition to the concerns regarding the specificity of AICAR as a pharmacological tool to study AMPK, concerns may arise by the use of Compound C, since it has been reported to inhibit AMPK as well as other protein kinases. However, we believe that our results obtained with trout myotubes using this inhibitor can be attributed to the inhibition of AMPK activity because Compound C completely reversed the stimulatory effects of AICAR (which is not known to target other protein kinases) on glucose uptake, a condition considered evidence for a role for AMPK [47], as well as the effects of metformin, and even phenformin (data not shown), on glucose uptake. In support for the use of Compound C in studying the role of AMPK in skeletal muscle cells, several studies have reported identical results when blocking AMPK activity with Compound C and when decreasing AMPKα subunit levels by siRNA in mammalian cells [44], [54].

**Figure 10:**
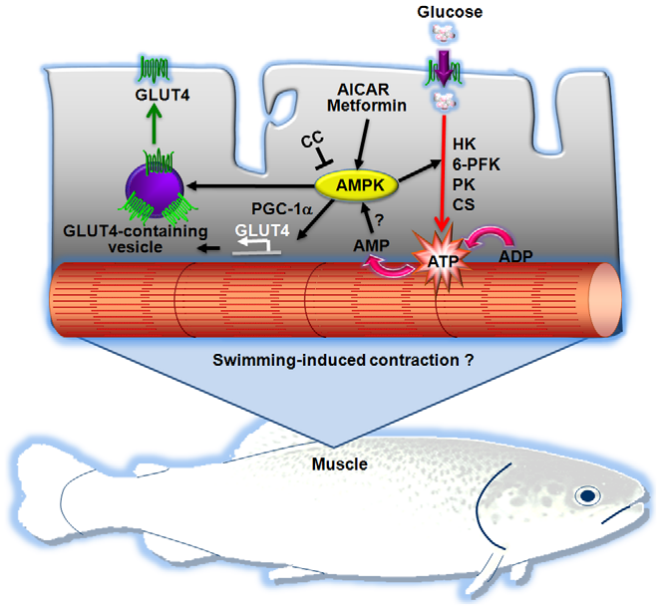
Proposed model for the role of AMPK in fish skeletal muscle cells. We propose that activation of endogenous AMPK by pharmacological activators (i.e. AICAR or metformin) results in increased glucose uptake by fish skeletal muscle cells through two mechanisms involving GLUT4: 1) increased mRNA levels of GLUT4 (possibly through the increased expression of peroxisome proliferator-activated receptor γ coactivator 1α or PGC-1α) and 2) stimulation of the translocation of GLUT4 to the plasma membrane. Furthermore, we propose that pharmacological activation of AMPK may also increase glucose utilization by stimulating expression of hexokinase (HK), 6-phosphofuctokinase (6-PFK), pyruvate kinase (PK) and citrate synthase (CS) mRNA levels. Future studies should be devoted to examine the possibility that AMPK may be activated as a result of the contraction of skeletal muscle fibers in swimming fish.

The translocation of GLUT4 from an intracellular location to the plasma membrane and transverse tubules is thought to be the major mechanism by which exercise, AMPK activators and insulin increase skeletal muscle glucose transport in mammals [55]. In the present study, we propose that the enhanced glucose uptake by AICAR and metformin in trout myotubes may be due to an increase in the cell surface levels of GLUT4, as suggested by our results using an L6 muscle cell line stably expressing brown trout GLUT4 [29]. This heterologous (mammalian) cell system, for lack of a homologous (fish) cell system, has proven to be a very useful model to study the traffic characteristics of brown trout GLUT4 in skeletal muscle cells [29], [34]. Although the increase in the cell surface levels of btGLUT4 by AICAR and metformin measured in our study was modest (approximately a 20% increase over controls), a similar mechanism involving increased GLUT4 translocation and, therefore, glucose uptake due to the activation of AMPK by AICAR and metformin, has been described in the mammalian muscle. In rats, AICAR administration causes a 1.5 to 2 fold increase of GLUT4 protein in the muscle [7], a 1.2 fold increase in the GLUT4 pool at the plasma membrane in the hindlimb [7] and a 57% increase in the sarcolemma to intracellular content ratio for GLUT4 in the myocardial muscle of AICAR-infused rats [56]. In rat cardiomyocytes, metformin has been shown to increase glucose uptake (5 fold) by increasing by 50% the retention time of GLUT4 at the plasma membrane [57]. Moreover, AICAR and metformin treatment provoked acute increases in the translocation of GLUT4 to the plasma membrane in mice gastrocnemius muscle and in L6 myotubes [37]. In addition to the increase in trout GLUT4 cell surface levels by AICAR and metformin, we show in this study that these two AMPK activators also increase GLUT4 mRNA levels in trout myotubes. Therefore, we believe that the observed increase in glucose uptake under the stimulus of AICAR and metformin in trout myotubes could be due, at least in part, to an increase in the cell surface and mRNA levels of GLUT4. In rats, a day after 6 h of swimming, GLUT4 expression in muscle was increased by 2 fold [58], whereas an *in vitro* incubation of rat muscle with 500 µM AICAR for 18 h increased GLUT4 expression by 1.5–1.6 fold [59], showing that AMPK activators enhance GLUT4 expression in this tissue. Interestingly, GLUT1 expression was not affected by AMPK activators in trout myotubes, suggesting that the observed increase in glucose uptake as a result of AMPK activation may be mediated primarily through GLUT4. A recent study examining the *in vivo* effects of metformin administration in rainbow trout indicated that this AMPK activator lowers blood glucose levels and increases GLUT4 expression in white skeletal muscle [33]. Our results on the direct stimulatory effects of AMPK activators on AMPK activity, glucose uptake and cell surface and mRNA levels of trout GLUT4 in skeletal muscle cells suggest that the reported hypoglycemic effects of metformin administration in rainbow trout [33] may result from the direct action of this compound on white skeletal muscle, increasing its ability to take up glucose through a mechanism involving AMPK activation and increased GLUT4 cell surface and mRNA levels (Fig. 10).

### AMPK activation may drive glucose towards the glycolytic pathway for energy generation

In skeletal muscle, once glucose enters the cells it is rapidly phosphorylated to glucose-6-phosphate (G6P) by HK. The fate of G6P is then two-fold: G6P can either be directed towards the synthesis of glycogen or, alternatively, G6P can be used for the direct generation of ATP by glycolysis and the TCA cycle. Our results show that pharmacological activation of AMPK by AICAR increased the mRNA levels of HK in trout myotubes, suggesting that activation of AMPK stimulates the entry as well as the metabolism of glucose in trout skeletal muscle cells, as it is the case in the mammalian muscle in response to exercise [55], [60]. Our results are also consistent with those of a recent study showing that *in vivo* administration of metformin in rainbow trout fed a high-carbohydrate diet increases the mRNA levels and activity of HK in white skeletal muscle [33]. Interestingly, our results also show that the glycogen content in trout myotubes was not affected by the AMPK activators AICAR and metformin and that the mRNA levels of GS were not affected by AICAR. Our results are consistent with studies in mammals that have shown that AMPK activation induces the phosphorylation of GS, decreasing its activity and, consequently, glycogen synthesis in skeletal muscle [61], [62]. However, other studies in mammals have reported the opposite effect, with AICAR stimulating allosterically GS by increasing the concentration of glucose-6-phosphate and, therefore, overriding the inhibitory phosphorylation of GS [63], [64]. Although there is no direct evidence in fish on the regulation of GS activity by AMPK activators, the inability of AMPK activators to alter glycogen and GS mRNA levels in trout myotubes suggests that pharmacological activation of AMPK does not result in the use of glucose for glycogen synthesis. In contrast, the stimulation by AICAR of the mRNA levels of 6-PFK and PK, two key enzymes in the glycolytic pathway, and of CS, an enzyme controlling one of the flux-determining steps of the TCA cycle, strongly suggests that AMPK activation in trout myotubes may turn on metabolic pathways that lead to the generation of energy from glucose. Therefore, in fish skeletal muscle cells, pharmacological activation of AMPK may result in increased glucose uptake and flux through glycolysis, as in mammals [65], rather than through glycogenesis (Fig. 10).

Although we did not measure changes in ATP concentration in trout myotubes in our experiments, there is strong evidence that cellular ATP levels are relatively stable in vertebrates, despite significant changes in pathway fluxes and energy provision to the muscle [66]. Therefore, the increase in glucose uptake and the lack of change in glycogen content produced by the activation of AMPK by AICAR and metformin suggest that an inhibitory mechanism may be present to avoid a build-up of ATP in the cell. Such mechanisms may include the allosteric down-regulation of AMPK activity by ATP levels [67], the dephosphorylation of the enzyme by protein phosphatases 2A (PP2A) and 2C [68], [69], [70] or its inhibition by the serine/threonine protein kinase Akt [71].

The mechanisms by which AICAR and metformin regulate the transcription of GLUT4 have been described in the mammalian muscle and may involve the phosphorylation of PGC-1α by AMPK [72], [73] and the binding of myocyte enhancer factor 2 to the GLUT4 promoter to increase the transcription of the GLUT4 gene [74], [75]. Additionally, recent studies suggest that exercise and pharmacological activation of AMPK also serve as important signals in regulating the expression of PGC-1α in skeletal muscle [76], [77]. PGC-1α is also involved in the transcriptional regulation of several genes implicated in the generation of ATP, mainly from oxidative metabolism [78], [79]. In particular, PGC-1α is known to mediate the stimulatory effects of AMPK on fatty acid oxidation in skeletal muscle [80]. In the present study we show that AICAR increases PGC-1α mRNA levels in trout myotubes (Fig. 9), which agrees with the described effect of this AMPK activator in the mammalian muscle [81], [82]. The observation that AMPK activation in trout myotubes results in increased PGC-1α and CS mRNA levels, coupled with recent data showing that exercise increases PGC-1α and CS mRNA levels in the skeletal muscle of zebrafish [83], suggest that AMPK could be involved in mediating the stimulatory effects of exercise on PGC-1α and CS expression in fish skeletal muscle. It will be interesting to determine in future studies if the pharmacological activation of AMPK in trout myotubes, or even *in vivo* conditions that may result in increased AMPK activity (e.g. swimming-induced exercise), could increase fatty acid oxidation.

### Conclusions and Perspectives

In summary, AICAR and metformin, two compounds that are effective stimulators of muscle AMPK activity in mammals, have been shown to stimulate AMPK activity and increase glucose transport in trout myotubes through a mechanism that appears to be mediated by GLUT4. Furthermore, AMPK activation in trout myotubes results in increased expression of key genes involved in ATP generation from glucose through glycolysis (Fig. 10). The results from the present study suggest that the metabolic role of AMPK in skeletal muscle appears to have been conserved throughout evolution from fish to mammals, evidencing the importance of AMPK in energy regulation in vertebrates. Furthermore, our observations provide essential information to test, in future studies, the hypothesis that AMPK may be a potentially important intermediary in the signaling cascade leading to contraction-stimulated glucose transport and utilization in fish skeletal muscle. Specifically, research must be conducted to determine whether AMPK plays an important metabolic and energy sensing role during swimming-induced exercise in fish.
